# And the subsidiary lives on: Harnessing complex realities in the contemporary MNE

**DOI:** 10.1057/s41267-022-00552-4

**Published:** 2022-07-29

**Authors:** Daniel S. Andrews, Phillip C. Nell, Andreas P. J. Schotter, Tomi Laamanen

**Affiliations:** 1 Ivey Business School, Western University, London, Canada; 2 Institute for International Business, Vienna University of Economics and Business, Vienna, Austria; 3 Department of Strategy and Innovation, Copenhagen Business School, Frederiksberg, Denmark; 4 Institute of Management & Strategy, University of St. Gallen, St. Gallen, Switzerland

**Keywords:** global strategy, international business, multinational corporation, structural differentiation, subsidiary management

## Abstract

Managing multinational enterprise subsidiaries is a core facet of international business research. A shifting reality on the ground has triggered concerns around the waning relevance of the subsidiary because the MNE and its structure and processes have become increasingly complex. Consequently, more decentralized, responsive, and fluid organizational designs are now at the core of IB research. Juxtaposing recent arguments questioning subsidiary research altogether, we argue that IB scholars can explore and explain complex realities in the contemporary MNE without unnecessarily restricting the breadth of the field and giving up links to established research and theory. We reframe conversations around inward- and outward-looking perspectives, providing a path forward that emphasizes the importance of embracing the subsidiary concept in research reflecting today’s complex business environment.

## Résumé

La gestion des filiales des entreprises multinationales (Multinational Enterprise - MNE) constitue une facette essentielle de la recherche en affaires internationales (International Business - IB). L'évolution de la réalité sur le terrain a suscité des inquiétudes quant à la perte de pertinence des filiales, étant donné que la MNE, sa structure et ses processus sont devenus de plus en plus complexes. Par conséquent, sont désormais au cœur de la recherche en IB les conceptions organisationnelles plus décentralisées, plus réactives et plus fluides. En juxtaposant les récents arguments remettant en cause la recherche portée sur les filiales, nous argumentons que les chercheurs en IB peuvent explorer et expliquer les réalités complexes des MNEs contemporaines sans restreindre inutilement l'étendue du domaine et sans renoncer aux liens avec la recherche et la théorie établies. Nous recadrons les conversations autour des perspectives autocentrées et orientées vers l’extérieur, tout en proposant une voie à suivre qui souligne l'importance d'inclure le concept de filiale dans la recherche reflétant l'environnement commercial complexe d'aujourd'hui.

## Resumen

La gestión de filiales de las empresas multinacionales (MNE por sus siglas en ingles) es una faceta central en la investigación en negocios internacionales. Una realidad cambiante en el terreno ha desencadenado preocupaciones sobre la disminución de la relevancia de la filial debido a que la empresa multinacional y su estructura y procesos se han vuelto cada vez más complejos. En consecuencia, los diseños organizacionales más descentralizados, receptivos y fluidos constituyen ahora el núcleo de la investigación sobre las empresas internacionales. Frente a los recientes argumentos que cuestionan la investigación sobre las filiales, sostenemos que los estudiosos de negocios internacionales pueden explorar y explicar las complejas realidades de las empresas multinacionales contemporáneas sin restringir innecesariamente la amplitud del campo ni renunciar a los vínculos con la investigación y la teoría establecidas. Replanteamos las conversaciones en torno a las perspectivas hacia adentro y hacia afuera, proporcionando un camino a seguir que subraya la importancia de adoptar el concepto de filial en la investigación que refleja el complejo entorno empresarial actual.

## Resumo

Gerenciar subsidiárias de empresas multinacionais (MNE) é uma faceta fundamental da pesquisa em negócios internacionais (IB). Uma realidade mutante no terreno desencadeou preocupações em torno da diminuição da relevância da subsidiária porque a MNE e sua estrutura e processos se tornaram cada vez mais complexos. Consequentemente, designs organizacionais mais descentralizados, responsivos e fluidos estão agora no cerne da pesquisa em IB. Justapondo argumentos recentes que questionam a pesquisa sobre subsidiárias como um todo, argumentamos que estudiosos de IB podem explorar e explicar realidades complexas na MNE contemporânea sem restringir desnecessariamente a amplitude do campo e abrir mão de relações com a pesquisas e a teoria estabelecidas. Reestruturamos conversas em torno de perspectivas que vislumbram o exterior e o interior, fornecendo um caminho a seguir que enfatiza a importância de adotar o conceito de subsidiária na pesquisa que reflita o complexo ambiente de negócios de hoje.

## Abstract (zh)

管理跨国企业 (MNE) 子公司是国际商务 (IB) 研究的一个核心方面。由于MNE及其结构和流程变得越来越复杂， 当地不断变化的现实引发了人们对子公司相关性减弱的担忧。因此， 更加去中心化、反应灵敏和流动的组织设计现在是 IB 研究的核心。我们将最近质疑子公司研究的论点并列在一起， 认为 IB 学者可以探索和解释当代MNE中的复杂现实， 而不会不必要地限制该领域的广度和放弃与既定研究和理论的联系。我们围绕内向和外向观点重新构建对话， 提供了一条前进的道路， 强调了在反映当今复杂商业环境的研究中采用子公司概念的重要性。

## INTRODUCTION

The multinational enterprise (MNE) subsidiary is a core facet of international business (IB) research. Extant literature highlights the importance of subsidiaries and their management, including their roles in entering foreign markets, building firm-specific advantages, and driving the overall success of the MNE (Meyer, Li, & Schotter, 2020). While the management of subsidiaries presents numerous challenges, they are the foundation of the MNE’s very existence. Yet, several scholars have recently voiced concerns about the phenomenological and methodological issues that allegedly plague extant subsidiary scholarship. Most recently, an article by Edwards, Svystunova, Almond, Kern, Kim, and Tregaskis (2021) highlighted the potentially eroding relevance of the subsidiary concept, arguing that its existing applications and, thus, subsidiary-focused research questions in IB are outdated and problematic.

The expressed waning relevance of the subsidiary comes from a presumed shifting reality on the ground. Edwards et al. (2021) argue that subsidiaries are an increasingly rare phenomenon primarily because the term is seldom used in practice. They further argue that the subsidiary-based assumptions of substantial authority and control, as well as a clear country focus, are conceptually inappropriate. Therefore, they call for more exploratory and qualitative research to capture the various forms of cross-border interaction, fluidity, and contestation in MNEs today; they also suggest using an institutional lens, such as the work on dynamic institutional fields. Although we share some of the authors’ general concerns, we depart from their portrayal of subsidiaries as “close to meaningless” (Edwards et al., 2021: 4) and, consequently, their methodological and theoretical recommendations as well.

First, Edwards and colleagues (2021) present an ill-defined caricature version of a subsidiary; it is an easy target, and they assault this. However, well-established IB research acknowledges the prominence of decentralized, responsive, and fluid organizational designs (Bartlett & Ghoshal, 1989), delineating the various roles among lower-level actors that matter in the cross-national space where activities occur. Recent studies also leverage various analytical techniques to model complex realities without abandoning the subsidiary concept (Andrews, Fainshmidt, Gaur, & Parente, 2021; Phillips, Petersen, & Palan, 2021; Tippmann, Scott, & Parker, 2017). Ignoring well-established as well as more recent research does not foster sincere scholarly discourse; addressing the “structures and processes that matter” does not require an unnecessary bifurcation of the literature. A contemporary conceptualization of the subsidiary fits within the parameters of traditional theory and research.

Second, COVID-19 – and Brexit and the Russian invasion of Ukraine – paired with a rising general skepticism toward globalization, has highlighted the relevance of subsidiaries as legal entities within formal MNE hierarchies, as well as the importance of local identity and legitimacy. Now more than ever, organizations are responding to new realities in the global economy by deepening their commitments to their various national contexts and responding to the pressures of their local stakeholders (Ciravegna & Michailova, 2021) or, in the case of Russia, MNEs and their subsidiaries are abruptly exiting due to local pressures (Colten, 2022). In the post-pandemic economy, “MNCs and their subsidiaries have the opportunity to demonstrate the mutually beneficial outcomes for the home and host countries, and thereby contribute to the re-emergence of co-operative national policies” (Meyer & Li, 2022: 18). Indeed, these developments slow down or reverse some of the dynamics underlying Edwards et al.’s (2021) alleged erosion of the subsidiary concept.

This article emphasizes the need to embrace subsidiaries in future international business research. We argue that IB scholars are well accustomed to exploring and explaining complexities in MNEs and can continue to do so without unnecessarily departing from established theory and research. The dynamics in today’s business environment are giving new meaning to subsidiary research as well. Hence, we caution against a narrow “subsidiary-free” approach as a corrective measure: research can identify, model, and deal with complexities in the contemporary MNE by leveraging decades of research and novel theoretical and empirical apparatuses.

In the following sections, we revisit Edwards and colleagues’ (2021) arguments and their value to changing IB research conversations. We explain why subsidiaries still matter and why they may matter even more in the coming years. We then characterize subsidiary research using a multiplicity, multiplexity, and dynamism framework to identify those challenges IB researchers may face in addressing subsidiary-relevant phenomena. We conclude by introducing inward and outward-looking solutions to accommodate subsidiary-relevant complexities in future research, advancing a scholarly agenda to study the contemporary MNE and its subsidiaries today.

## AN ASSESSMENT OF EDWARDS ET AL. (2021)

### Key Arguments

Edwards et al. (2021) document several concerns about the prevalence of the term subsidiary in IB research while referring to a subsidiary as an organizational unit with substantial authority, control, and country focus. The authors do so by questioning the traditional assumptions of MNE structures and processes “from above” and “from below.” The first concern highlights the broad range of global strategies and structures contemporary MNEs pursue in dynamic global markets. Specifically, MNEs increasingly organize themselves into international divisions, which may be supplemented by cross-national structures, to achieve global efficiency through scale. These MNEs cohere around core activities rather than national contexts, making functions the primary axis of the organization. They assert that it is feasible that multiple functions reside in each locality without an overarching national subsidiary structure and vary considerably in the locus of control.

The second concern emphasizes the microlevel developments that have emerged from below the national level. As the nature of work becomes more dynamic, interactions among employees increasingly occur on a global scale rather than in each national context. Digital technologies now shape how employees interact and with whom they interact most frequently. Especially during periods of environmental turbulence and upheaval, MNEs may use virtual teams and various mechanisms to reconfigure their organizations and drive efficiencies (Caliguri, de Cieri, Minbaeva, Verbeke, & Zimmerman, 2020). The result is an organization where more globalized actors differentially engage in cross-national networks, occupy various positions, and are embedded in different control structures (Schotter, Maznevski, Doz, & Stahl, 2021).

The combined implications of these trends are argued to “fundamentally change the ways in which we think of MNCs’ local presence” (Edwards et al., 2021: 4). Consequently, the authors omit the subsidiary altogether and assert strong recommendations regarding the phenomena that IB researchers should study – or for that matter, not – and the methodological and theoretical approaches they should use. Specifically, they narrowly argue that exploratory, qualitative work on dynamic institutional fields is a promising avenue for future research because the national context is not the primary domain of some subsidiaries. Below we argue why this chain reaction is far-reaching and flawed.

### Criticisms and Missed Opportunities

While we echo some of Edwards et al.’s (2021) points, most of their criticisms are exaggerated or ill founded. For instance, their criticisms based on the assumption of country focus and locus of authority and control are already reflected in a more relaxed definition and conceptualization of subsidiaries established more than two decades ago. More specifically, Birkinshaw, Hood and Jonsson (1998: 224) succinctly suggest that subsidiaries in MNEs are “any operational unit controlled by the MNC and situated outside the home country”. While being critical of extant work is welcomed, there is little to gain by derailing decades of subsidiary research that can still be leveraged to explain MNE and subsidiary-relevant issues today.

Our review of Edwards et al. (2021) culminated in three key issues and missed opportunities. First, the criticisms may seem appropriate in assuming a narrow set of subsidiary-focused work, perhaps reflecting a miniature replica of the corporate headquarters (Birkinshaw & Morrison, 1995). These subsidiaries would have a wide range of authority or control over all essential business functions and activities, focus on one single host country, and be nearly stand-alone, enterprises. However, Edwards et al. (2021) are notably vague as they do not sufficiently define what a subsidiary is in their view; the critique is built loosely on assumptions that prior research often differs from (Meyer et al., 2020). The strawman tactics and absence of a differentiating definition are highly problematic because decades of subsidiary research have demonstrated that subsidiaries vary across the activities they perform (Meyer et al., 2020), the decision rights they possess (Frost & Birkinshaw, 2002), the power they hold versus headquarters (Bouquet & Birkinshaw, 2008), and the geographic regions they serve. The latter, in particularly, includes subsidiaries with subnational (Ma & Delios, 2010) or supranational mandates such as regional headquarters (Pla-Barber, Botella-Andreu, & Villar, 2021) or centers of excellence (Frost et al., 2002). Recent studies have further extended these insights to today’s organizations, for example, by examining subsidiary roles in emerging market hybrid organizations (Ambos, Fuchs, & Zimmerman, 2020), subsidiaries’ cross-functional, cross-border reporting lines (Ambos, Kunisch, Leicht-Deobald, & Steinberg, 2019), and within-MNE and within-subsidiary control differences (Dattée, Arrègle, Barbieri, Lawton, & Angwin, 2022). Indeed, these insights have all surfaced without an undue change in nomenclature.

Edwards et al.’s (2021) concerns are at least partly motivated by the ongoing digital transformation of organizations. Technology has enabled firms to modularize work and improve the efficiencies of coordinating complex activities across functional and geographic areas (Luo, 2022). Digitalization may reduce the need for a “national subsidiary” because work is not location bound, and employee interactions are more fluid. Digital, born-global firms have been the pioneers of these shifts (Birkinshaw, 2022). However, these firms started with different principles and should not be equated to the traditional MNEs on which most subsidiary research has focused. Even those MNEs that manage to adopt new ways of organizing often must resort to the use of formal subsidiary structures to manage their existing international operations. For example, SAP structures its Canadian operations around a Toronto subsidiary (SAP Canada), which oversees independent research and development “labs” in Montreal, Vancouver, and Waterloo. These labs have their own work forces and projects, such as building products for global distribution in collaboration with other units worldwide, while still being subject to the oversight of the Toronto subsidiary and the rules and regulations of the Canadian federal and provincial legal systems. The formal subsidiary structure also shapes the legitimacy and local identity of the MNE in the host country (Lee, Kim, & You, 2022; Pant & Ramachandran, 2017). Thus, it is erroneous to assume that MNE structures and processes today are suddenly more fluid with few concessions to legacy structures.

Second, Edwards and colleagues (2021) neglect legal issues entirely. The MNE is a de facto network of legally registered subsidiaries that individually own assets and partake in arm’s-length contracts within and outside the firm (Phillips et al., 2021). Subsidiaries are subject to country-specific policies and laws – such as jurisdictional rules and regulations – regardless of their heterogenous mandates and processes. Corporate law asserts that a national subsidiary must have formally appointed management who bears the legal responsibility for the local entity (Robé, 2011). While a subsidiary may, in some cases, be a mere fictional shell to optimize tax benefits (Barrera & Bustamante, 2018), these legal entities and the persons responsible for them are liable for their actions. As MNE and government relationships are increasingly central to understanding structure, strategy, and performance (Miroudot, 2020), we argue that the legal identification of a subsidiary and its management as separate entities is now more critical than ever, not less so.

The legal separation of a subsidiary also highlights governance issues, such as the orchestration of units to engage in transfer pricing, profit sharing, legal portioning, sequencing, and corporate arbitrage (Karayan, Sweden, & Neff, 2002). For instance, tax optimization strategies have become increasingly commonplace; MNEs use national subsidiaries to implement sophisticated ways to take advantage of various institutional systems (Eicke, 2009). These strategies are seldom conducted within a single subsidiary, so MNEs need a deep understanding of individual units within the corporate portfolio in order to govern them, and so do IB researchers to study them. A German subsidiary may conduct all regional operations for a product line but show little or no profit to optimize its tax liabilities; the obligation may be transferred to a Cayman Islands or Bermudian subsidiary where there is a lower tax obligation. The governance of national subsidiaries as separate legal entities located in different jurisdictions spotlights these potential differences in MNE strategy (Phillips et al., 2021). Hence, another key implication is that national differences in tax policies, schemes, and regulations make subsidiaries an essential object of study in contemporary MNEs.

Finally, even if one would accept that the new ways of organizing have contributed to the erosion of the single-country-focused subsidiary concept in the globalized environment, lessons from the post-pandemic global economy and increasing skepticism of globalization (Cuervo-Cazurra, Doz, & Gaur, 2020) may undermine some of these dynamics. Specifically, the globalization of MNE structures and processes has led to a counterreaction and an increasing interventionist role of the state (Ciravegna & Michailova, 2021). Consequently, MNE managers must justify the relative benefits they bring to each country in which they operate, regardless of their subsidiary’s local product and operational mandates. The country context may, in some cases, turn less favorable as well, creating constricting business environments through increased regulations and policies. MNEs may pull out of these less fertile host countries to maintain salience and long-term viability under ambiguity, such as recent departures from Russia and, to a lesser extent, China and the United Kingdom (Brexit). The broader notion is that MNEs and their subsidiaries need to possess well-developed relationships with increasingly influential stakeholders, deepening coordination among economic and political actors (Lawton, Dorobantu, Rajwani, & Sun, 2020) and putting legitimacy and identity at the forefront of their strategy (e.g., Holm, Decreton, & Nell, 2017). Recent research suggests that these developments may even result in subsidiaries being granted even more operational autonomy than before (Meyer & Li, 2022), directly contradicting the purported “unease” about authority and control residing at the subsidiary level.

To be clear, we agree that the field should investigate in more detail how MNEs work and how they are structured, which indeed means getting one’s hands dirty. But neglecting existing terminology is unjustified in practice and would also unnecessarily restrict the breadth of our field and give up links to established theory. The subsidiary term allows researchers to signal that their work belongs and builds on decades’ worth of subsidiary management research within MNEs (Meyer et al., 2020). It also allows the field to maintain an overview of the manifold contributions and to judge if and how new contributions contribute to the field’s cumulative insights. Even if the term may be used less frequently in practice, subsidiaries are still the legal and organizational foundation of MNEs’ existence and evolution; understanding their evolution is even more critical in the post-pandemic global economy.^1^ Thus, the claims that we should eliminate subsidiary-focused research and instead allocate scholarly attention entirely to new theory development around multiple forms of cross-national interactions – which are allegedly more fluid and less formal – seems unsubstantiated. We argue that the attempt to “whither national subsidiaries” is a case of false uniqueness (Aguinis & Gabriel, 2021).

## CHARACTERIZING COMPLEXITIES IN SUBSIDIARY RESEARCH

While we have criticized the conclusions of Edwards et al. (2021), we acknowledge that the structures and processes of MNEs and subsidiaries are complex – or perhaps less so in the future, given ongoing socio-political shifts – and present numerous managerial and practical challenges. And as with any research domain, such research needs continuous updating. However, rather than steering future research beyond the subsidiary sphere, we argue that future IB research could benefit from a characterization of complexities to identify the challenges of addressing subsidiary phenomena. Hence, this section briefly examines the extant subsidiary research to characterize its complexities. We distill complexity into three parts to illustrate what it is and what it looks like in the subsidiary context. Our characterization lucidly identifies extant opportunities and challenges in a well-documented research area.

### Interactions among Actors over Time

Prior research focuses on many different actors, levels, and outcomes, suggesting an inherent complexity to the questions and phenomena considered by subsidiary-focused scholars (Meyer et al., 2020). These complexities have implications for the theoretical (Bello & Kostova, 2012) and methodological (Eden & Nielsen, 2020) approaches leveraged to motivate research, analyze questions, and frame contributions.

The first two sources of complexity – *multiplicity* and *multiplexity* – refer to the interactions among the actors that matter. More specifically, multiplicity captures the number and variety of actors, such as employees, managers, industries, and countries that create opportunities and challenges for advancing subsidiary research. For instance, it is well documented that a subsidiary is simultaneously embedded in its local industry and organizational hierarchy, as well as exposed to other contextual factors, such as city-regions, which may shape its structures and strategy (Meyer, Mudambi, & Narula, 2011). Subsidiaries may also have many different functions (e.g., accounting, human resources, information technology) within a national subsidiary structure, shaping the importance of any given actor for a particular functional area; many actors can influence subsidiary phenomena within or outside the MNE and on higher or lower levels of aggregation than at the subsidiary level. Hence, a conceptualization of the combined effects of the many actors may be necessary to conceptualize subsidiary-relevant phenomena accurately.

The second source of complexity is the *multiplexity* of interactions among the multiplicity of actors. Eden and Nielsen (2020: 1613–1614) argue that “multiplexity is created when there are “networks of networks,” generating systemic problems such as cross-level effects, feedback loops, diffusion, and contagion.” Of particular interest are the structures and systems of routines and workflows embedded in geographically dispersed and functionally distributed subsidiaries. These complexities closely resemble the work of Nohria and Ghoshal (1994) more than two decades ago. For instance, subsidiary structures and processes vary based on the trade-offs between the challenges and opportunities presented in the home and host markets and how the unit and its various internal activities interact and shape the processes to identify, generate, and assimilate resources and capabilities (Andrews et al., 2021). Cantwell and Mudambi (2005) illustrate how these actors interact to transform the subsidiary from competence-exploiting to competence-creating mandates. Thus, subsidiary phenomena are often explained by how the demands of the external environment and the internal activities of the MNE and subsidiary influence each other.

The final source of complexity relates to *dynamism* or the evolution of MNE systems over time. Dynamism is prevalent in that trends, business cycles, and crises will continuously shape the structure and interactions of the MNE and its subsidiaries. The COVID-19 pandemic and the geopolitical tensions caused by the war in Ukraine have represented significant turning points for many MNEs. Some subsidiaries are abruptly exiting their host countries, as the local environments have turned less generative. This stresses the importance of acknowledging historical patterns among the relevant internal and external actors (Birkinshaw & Hood, 1998); for example, the exposure to longstanding political conflict and military action shapes subsidiary strategy and, ultimately, survival (Dai, Eden, & Beamish, 2013). Further, engaging with historical patterns allows a researcher to avoid the spurious labeling of a finding as new and allows researchers to include other factors that may explain the behavior and structuring of MNEs and their subsidiaries.

Importantly, the three dimensions of complexity are not mutually exclusive. The subsidiary must ensure it pays sufficient attention to its many stimuli – such as the specificities of internal and external actors (Andrews, Fainshmidt, Ambos, & Haensel, 2022) – regardless of whether a specific geographic scope remains or a broader or narrower mandate exists. As MNEs and subsidiaries evolve, the dynamics among these actors do, too (Cuervo-Cazurra, Mudambi, & Pedersen, 2019). An MNE may move from hierarchical to decentralized or a subsidiary from competence-exploiting to competence-creating depending on the nature of the external environment and the resources and capabilities accumulated or depleted. Thus, the three sources of complexity are often required to understand the full range of MNE- and subsidiary-relevant phenomena today, but they do not necessitate abandoning the subsidiary concept.

At the same time, it is not clear if all dimensions of complexity always matter. For instance, a subsidiary may have limited attachments to many stakeholders by nature of its mandate, or a nascent subsidiary may be shielded from some historical patterns within the MNE (Birkinshaw & Hood, 1998). Thus, the multiplicity of actors, for instance, may take a different form or level of importance given the characteristics of the subsidiary under scrutiny. These issues raise their own challenges related to conceptual boundaries, theory, and methodologies, which we discuss below.

## RESEARCH AGENDA: INWARD AND OUTWARD-LOOKING

Our brief overview of the extant subsidiary research uncovers several complexities relevant to IB research. Edwards and colleagues (2021) argue that these complexities often lead to shortcomings that plague scholarly progress, and are primarily the result of literature not relaxing assumptions enough. While using an overarching term, such as subsidiary, is, at times, a (warranted) simplification of complex realities, this claim seems unsubstantiated given the considerable volume of existing research we have referred to thus far, as well as the emerging trends in the global economy.

Furthermore, assumptions may be vital to advancing a broader research domain. Because research involves “representing real phenomena in ‘stylized’ or ‘idealized’ ways” (Foss & Hallberg, 2014: 903), assumptions are important insofar as they determine how proximate a study and its theory are to reality. Assumptions can help isolate one of the three sources of complexity while providing tractability and concentrated focus on specific mechanisms or theoretical isolation. Indeed, empirical and theoretical apparatuses, such as experimental designs (e.g., Cerar, Decreton, & Nell, 2022), simulations (e.g., Celo & Lehrer, 2022), and game theory (e.g., Asmussen, Foss, & Nell, 2019), as well as purely theoretical pieces (e.g., Kostova, Nell, & Hoenen, 2018), often work with simplifying assumptions. Still, these approaches have contributed to our cumulative understanding of how various organizational actors interact and shape structures and processes that matter. While there will always be disagreement regarding what assumptions are too extreme and essential or fundamental to the specific phenomenon under scrutiny, these debates are critical – assumptions matter in devising research questions, selecting theories, and deploying methodologies.

In this section, we provide direction for future subsidiary-focused research that emphasizes the need for tractability as well as an embrace of complex realities. We do so by offering a way forward that focuses on clearly stating the boundaries of concepts, questions, and analyses to more clearly understand the limits of a given research design to answer a given research question. We identify opportunities to refine and expand theoretical and empirical approaches to overcome blind spots and advance research into new areas, such as the changing role of subsidiaries in the post-pandemic world.

Our guiding argument is twofold. First, given the complexity of the subsidiary context, future research needs to define and delineate concepts clearly and justify their relevance. Second, while aiming to gradually capture more of the extant complexity in subsidiary research, research should strive for alignment between research questions, theory, and methodological approach. Table 1 summarizes our suggestions, which are meant to be illustrative toward this end rather than aiming to point to a limited set of research topics.^2^

**Table 1:** Advancing a contemporary subsidiary research agenda

	Overall suggestions	Exemplary guiding questions with regard to …		
		Multiplicity of actors	Multiplexity of interactions	Dynamism
Questions and concepts	Clearly define and delineate the subsidiaries of interest Make assumptions explicit and highlight the limitations Spotlight dimensions of complexity before choosing a subsidiary-related research question	What types of subsidiaries matter and how can they be defined in terms of authority, control, and geographic focus? What other actors matter, and how and why do they matter? Should the study focus on one type of subsidiary or not? How does the study context or recent developments inform the boundaries of the subsidiary concept?	Which interactions between actors matter? How do cross-level effects and feedback loops shape subsidiary structures and processes? How does the interconnectedness of MNE operations inform the boundaries of the subsidiary concept?	How do emerging trends, such as global disruptions and environmental risk and uncertainty, change authority and control within subsidiaries? How does the stage of the subsidiary development change the relevance of the research questions being examined?
Theoretical applications	Understand and discuss the applicability of theories and how they vary given contextual influences and boundaries of the subsidiary Avoid ecological fallacies where a theory developed for one level is used at a different level inappropriately	How do core theories, such as agency, institutional, and resource dependence theories, differ from the perspective of the MNE or subsidiary? How do lower-level actors interact with and shape subsidiary phenomena?	How can IB researchers contextualize existing theories to account for the impacts of many actors? How can multiple theoretical perspectives be combined to accommodate the plurality of interactions within the MNE?	Are specific theories less appropriate during phases of a subsidiary’s evolution? What are the boundaries of core theories in explaining subsidiary phenomena during dynamic exogenous shocks (e.g., pandemics, wars, environmental disasters)?
Methods	Carefully select the methodological approach that captures and deals with complexity, which might include deliberate and justifiable simplifying assumptions Address within-sample heterogeneity and possible nonequivalence Perform multiple robustness tests for subsamples Triangulate data sources	When should individuals, teams, or functions be included in multilevel models? How can IB researchers simultaneously consider relevant positive and negative aspects of country conditions?	How can IB researchers use structural models to understand control pressures from multiple levels within the MNE? How do the interactions and compositions of external and internal actors affect subsidiary-level phenomena?	How can mixed methods avoid the simplification of subsidiary complexities? What findings should be subject to replication across contexts and sample periods? How does time take a different connotation given exogenous shocks?

### Questions, Concepts, and Assumptions

Much IB research involves simplification and abstraction to signal that a given work belongs to a specific field and propose that the findings are generalizable beyond a specific context. However, it is equally relevant to explicitly account for contextual differences that allow researchers to create new theories or falsify or modify previous studies’ insights (Dau, Santangelo, & van Witteloostuijn, 2021).

Most critically, we believe future subsidiary research needs to define and delineate the boundaries of the subsidiary concept clearly, such as its geographic scope and mandate(s), its structural position within the MNE, and its legal form. For example, a subsidiary can have an international, subnational, national, regional, or city focus (Cantwell & Mudambi, 2005) or several of these simultaneously. Subsidiaries might cover a broad range of activities or focus on some more selectively, such as on R&D (Asakawa, 2001), manufacturing (Nell & Ambos, 2013), or marketing and sales (Edwards, Ahmad, & Moss, 2002), which may shape the implications of the analyses. Such contextualization helps highlight the boundaries within which theories are applicable; these distinctions should not only be made in the exposition of the study’s data. Further, an explicit focus on sources of complexity – for example, whether the subsidiary is an element in a complex transfer pricing structure (Phillips et al., 2021) – can help clarify underlying assumptions and discern why, how, and when the subsidiary concepts are or are not equivalent across contexts. Thus, we argue that a productive approach is to define clearly what is meant by the subsidiary concept in each research design, rather than merely debate “whither national subsidiaries” or not.

In some settings, these efforts may require that scholars more narrowly define their research questions. For example, a long-standing body of IB research argues that institutional distance may influence the performance of MNEs and their foreign subsidiaries (Kostova et al., 2019). Yet, national-level institutional distance may be irrelevant in some settings. Some subsidiary functions may rely more on subnational or city-specific actors that influence local structures and processes (Lorenzen, Mudambi, & Schotter, 2020). Especially in the post-pandemic world, subsidiaries have a heightened awareness and responsibility to local stakeholders. Thus, analyzing cross-national institutional differences may be less useful, and the multiplicity of host country actors may be more important in understanding subsidiary behavior and performance. This does not imply that cross-national differences are less relevant, but rather the extent to which they provide a realistic understanding of subsidiary processes will depend on the conceptual boundaries set in the research design. A narrower focus can help break new ground or extend existing knowledge bases. Hence, the selection of research questions should primarily reflect the sources of complexity that matter, driving data collection efforts. Further efforts in this direction would bring research closer to phenomenological accuracy and contain overexaggerated findings.

### Theoretical Applications

Subsidiary scholars often draw on theories without significant adaptation across contexts and organizational types. This is likely the result of formulating a study’s narrative to fit existing conversations. However, understanding the applicability of theories and how their mechanisms vary across contexts is central to avoiding fallacies, such as when a theory was developed for one level but inappropriately applied at a different level. For instance, applications of the attention-based view are often aggregated at the organizational level, overlooking the role of the attention seeker and attention giver (Andrews et al., 2022). However, the allocation of attention in an MNE is a dynamic multilevel process that unfolds through the interplay of individual actors and formal organizational structures (Ocasio, Laamanen, & Vaara, 2018). Similar to how the headquarters maintains some degree of formal control and authority over its dispersed and disaggregated units (Nell, Kappen, & Laamanen, 2017), the formal subsidiary structure can play an important symbolic role in employees’ motivation and focus of attention. Of particular theoretical interest is how these processes unfold in the era of deglobalization as subsidiary managers assume greater responsibility for their units but have reduced access to corporate resources (Meyer & Li, 2022). Here, the emphasis is on a sounder application of theoretical apparatuses as a necessary condition to improve contributions and push research frontiers (Bello & Kostova, 2012).

The need to add nuance is important, especially in the post-pandemic economy. Scholars can do so by drawing from adjacent fields, such as strategic management and organizational behavior, or deploying multiple theoretical perspectives simultaneously. For example, we can invoke the behavioral theory of the firm to provide a more dynamic view of the subsidiary comprised of a coalition of key actors – managers, employees, corporate functions, and customers – of which each has its own goals and expectations (Surdu, Greve, & Benito, 2021). These goals and expectations interact and evolve, and subsidiaries will vary in how they allocate resources to specific goals and, thus, the activities they undertake, structures they exhibit, and processes they manage. Recent developments in the global economy have spotlighted subsidiaries’ vulnerability to risks; this exposure shapes how they will behave in the coming years (Singh & Gaur, 2021).

To study countervailing forces in subsidiaries, there are also opportunities to simultaneously combine multiple theoretical perspectives. For example, Cuervo-Cazurra and colleagues (2019) integrate agency and resource dependence theory to better understand the locus of power and, thus, decision-making authority within the MNE. Because MNEs are differentiated networks with more fluid interactions, invoking complementary theories may be one means of providing a more thorough understanding of fluidity and differentiation.

### Methodological Toolkit

IB research has, over time, developed a sophisticated toolkit to deal with complexity head-on (Eden & Nielsen, 2020). Empirical tools such as fuzzy-set qualitative comparative analysis (Fainshmidt, Witt, Aguilera, & Verbeke, 2020) and structural equation modelling (Hult, Ketchen, Cui, Prud’homme, Seggie, Stanko, & Cavusgil, 2006) are relevant because they can model an appropriate combination of structural and processual elements, including complementarities and substitution effects. These approaches accommodate trade-offs imposed by each actor and, thus, help address the complex, multifaceted nature of those wicked problems facing organizations globally. For example, subsidiaries may introduce initiatives in response to changing environmental conditions. Yet, adopting these initiatives, or the performance of these units more broadly, may depend on the track record and credibility of subsidiary managers and the locus of control, as well as institutional conditions (Geleilate, Andrews, & Fainshmidt, 2020). Also, recent studies have leveraged network analysis (Li & Bathelt, 2020), multilevel contingency analyses (Stoermer, Davies, & Froese, 2021), and in-depth case studies (Ambos et al., 2020) to probe these complexities.

We also foresee an opportunity to accommodate the temporality of events through sequence analysis (Caren & Panofsky, 2005) and longitudinal panel data designs (Gaur, Pattnaik, Singh, & Lee, 2022), among others. Future research could use such techniques and data, for example, to explore the asymmetric and equifinal pathways to various MNE- and subsidiary-relevant outcomes given heterogeneous pre-pandemic conditions and response efforts. Subsidiary scholarship would also benefit from isolating a particular source of complexity. Li and Bathelt (2020) examine the knowledge strategies of foreign subsidiaries, arguing that the use of a given knowledge strategy depends on the clustering of actors and the subnational regional in which a subsidiary operates. In general, considering the multiplicity of internal and external actors involved in knowledge processes helps better illuminate subsidiary structures and processes (e.g., Mees-Buss, Welch, & Westney, 2019). Hence, we argue to preserve the concept of subsidiaries because existing empirical apparatuses can be deployed to craft powerful, complex insights.

## CONCLUSION

This article offers an alternative view to Edwards and colleagues’ (2021) critique of subsidiary research. We argue that explicitly recognizing complexities and accommodating them does not require abandoning the subsidiary concept and the large body of previous research. National subsidiaries often play important legal and symbolic roles in shaping organizational behavior, even if they are no longer the dominant subsidiary type. Using the subsidiary construct allows the field to position new contributions in relation to the cumulative insights of the field. We can further accommodate complexities in contemporary MNEs through existing and emerging theoretical and empirical apparatuses by building on the extensive body of literature that already exists. These efforts are particularly important in the post-pandemic economy where decades of research may provide initial clues for study and managing the subsidiary.

Hence, “*whither national subsidiaries*?” We firmly say no. We are also uneasy with narrow recommendations to focus on qualitative methods and institutional theory. Instead, IB research is well suited to tackle complex real-world issues through various theoretical and empirical lenses. This requires precise reasoning about why and which subsidiary concepts are relevant, how they are captured, how they fit the theoretical framing, and what research methods enable the identification of robust findings. We hope our commentary helps move scholarship on the contemporary MNE and its subsidiaries forward by building on, rather than abandoning, our extensive knowledge of subsidiaries.

## Notes

1. Some firms refer to subsidiaries as country units, network units, affiliates, or simply offices. The nomenclature notwithstanding, all labels refer to a lower-level organizational unit within an idiosyncratically defined organizational hierarchy.
2. We direct readers to more comprehensive reviews for specific, domain-related research questions (Kostoova, Marano, & Tallman, 2016; Meyer et al., 2020; Nell, Kappen, & Laamanen, 2017; Pla-Barber et al., 2021).
